# Development of a microinjection system for RNA interference in the water flea *Daphnia pulex*

**DOI:** 10.1186/1472-6750-13-96

**Published:** 2013-11-05

**Authors:** Chizue Hiruta, Kenji Toyota, Hitoshi Miyakawa, Yukiko Ogino, Shinichi Miyagawa, Norihisa Tatarazako, Joseph R Shaw, Taisen Iguchi

**Affiliations:** 1 Okazaki Institute for Integrative Bioscience, National Institute for Basic Biology, National Institutes of Natural Sciences, 5-1 Higashiyama, Myodaiji, Okazaki, Aichi 444-8787, Japan; 2 Department of Basic Biology, Faculty of Life Science, Graduate University for Advanced Studies (SOKENDAI), 5-1 Higashiyama, Myodaiji, Okazaki, Aichi 444-8787, Japan; 3 Environmental Quality Measurement Section, Research Center for Environmental Risk, National Institute for Environmental Studies, 16-2 Onogawa, Tsukuba, Ibaraki 305-8506, Japan; 4 School of Public and Environmental Affairs, Indiana University, 1315 East Tenth Street, Bloomington IN 47405, USA

**Keywords:** *Daphnia pulex*, Microinjection, RNA interference, *Distal-less*, Parthenogenetic egg

## Abstract

**Background:**

The ubiquitous, freshwater microcrustacean *Daphnia pulex* provides a model system for both human health research and monitoring ecosystem integrity. It is the first crustacean to have a well annotated, reference genome assembly that revealed an unusually high gene count highlighted by a large gene orphanage,-i.e., previously uncharacterized genes. *Daphnia* are capable of either clonal or sexual reproduction, making them ideally suited for genetic manipulation, but the establishment of gene manipulation techniques is needed to accurately define gene functions. Although previous investigations developed an RNA interference (RNAi) system for one congener *D. magna*, these methods are not appropriate for *D. pulex* because of the smaller size of their early embryos. In these studies, we develop RNAi techniques for *D. pulex* by first determining the optimum culture conditions of their isolated embryos and then applying these conditions to the development of microinjection techniques and proof-of-principle RNAi experiments.

**Results:**

We found that isolated embryos were best cultured on a 2% agar plate bathed in 60 mM sucrose dissolved in M4 media, providing optimal conditions for microinjections. Then, we injected double-stranded (ds)RNA specific to the *Distal-less* gene (*Dll*), which is a homeobox transcription factor essential for limb development in invertebrates and vertebrates. Injected embryos presented with defects in the second antenna and appendage development, and dsRNA induced the degradation of *Dll* mRNAs, indicating that this technique successfully inhibited transcription of the target gene.

**Conclusions:**

We developed a microinjection system for RNAi studies in *D. pulex*. These techniques add to the growing genomic toolbox and enhance the genetic tractability of this important model for environmental, evolutionary, and developmental genomics.

## Background

In both basic and human health research, there is a pressing need to understand how environmental conditions influence gene functions and, in turn, how individuals and populations cope with changing environments. Invertebrate species have emerged as models for experimental manipulation because of their unique biological attributes, life cycles, large numbers of offspring, easy maintenance, and sophisticated tools for high-throughput biology and open-source informatics [1]. However, the traits observed in laboratories are likely a small subset of the phenotypic variation that is expressed in natural ecosystems. This may partly explain why 15 to 50% or more genes are without experimentally determined functional annotations, even within the best-characterized genomes (e.g., yeast; [2]).

*Daphnia* possess several characteristics that make them valuable for environmental, evolutionary, and developmental genomics research–addressing the added complexity of genome-environment interactions. *Daphnia* are a ubiquitous, and ecologically important member of freshwater lakes and ponds, and have long been used as a sentinel of the integrity of these aquatic ecosystems. More recently with the release of the *D. pulex* genome [3], it now serves as a recognized surrogate model for human health research. The *D. pulex* genome possesses more genes than any previously sequenced animal genome (~31,000), due to a large orphanage of *Daphnia* genes that likely, allows the organism to respond to its environment [3]. In addition to their short generation time, large brood sizes, and ease of laboratory and field manipulation, *Daphnia* are capable of either clonal or sexual reproduction, making them ideally suited for genetic studies. At present, however, there are no effective methods for manipulating genes and characterizing gene function, which because of the large gene orphanage limits interspecies extrapolations.

RNA interference (RNAi) is an evolutionarily conserved post-transcriptional gene silencing mechanism, which is triggered by double-stranded (ds)RNA in a sequence specific manner [4,5]. Since RNAi was first reported in the nematode *Caenorhabditis elegans* by Fire et al. [6], it has been used as a powerful tool for the analysis of gene function in many organisms such as zebrafish *Danio rerio*[7], planarian *Schmidtea meditteranea*[8], cnidarian *Hydra magnipapillata*[9], fungus *Neurospora crassa*[10], fruit-fly *Drosophila melanogaster*[11] and mouse *Mus musculus*[12]. Microinjection is one method of introducing dsRNA into cells, and this method has been successfully developed for the daphnid species, *D. magna*[13]. In fact, microinjection techniques enabled the application of not only RNAi [13,14], but also overexpression of foreign genes [15] and the creation of transgenic individuals [16]. Establishing these techniques in *D. pulex* will extend the resources for environmental, evolutionary, and developmental genomics research for this species by providing needed tools to characterize gene function.

*Distal-less* (*Dll*) and its homologs *Dlx* genes, which function as homeodomain transcription factors, play one of the major roles in limb development throughout the animal kingdom [17]. Reduction of *Dll* activity caused defects of distal leg segments in arthropods including insects [18-20], crustaceans (*Parhyale hawaiensis*[21], *D. magna*[13]), the spider *Cupiennius salei*[22] and the spider mite *Tetranychus urticae*[23]. Because of its conserved role in limb development, defects in the expression of the *Dll* gene produce easily recognizable phenotype and ease of evaluation of its phenotype, which is why this endogenous developmental gene was selected as a target in proof-of-principle RNAi in *D. pulex*.

The goal of this study was to develop a microinjection system for RNAi in *D. pulex*. Culture requirements for isolated embryos were first determined and then microinjection techniques developed for conducting RNAi experiments using *Dll*-dsRNA.

## Results and discussion

### Development of microinjection system

There are two major technical problems for microinjection in daphnid species. One is the rapid hardening of egg membrane [24] and the other is a considerable difference between the internal and external osmotic pressures of the egg. The former prevents egg membrane penetration by a needle, and the latter produces leaking of internal contents (yolk, oil droplets, and so on) when the needle is withdrawn. Kato et al. [13] determined the proper condition for microinjection in *D. magna*. Eggs were incubated on ice just after ovulation to inhibit the membrane hardening transiently and placed in plastic petri dish with 80 mM sucrose medium dissolved by M4 culture medium (M4) to increase external osmotic pressure. However, these conditions are not suitable for *D. pulex* possibly because of the difference in egg size and form (Additional file 1). To overcome these hurdles, we first examined culture conditions by varying sucrose concentration and culture media, and determining those conditions that allowed embryos isolated from the brood chamber to develop normally. Survival was greater in M4 culture media as compared to dechlorinated freshwater (FW) (Table 1). A sucrose concentration of 40 mM on a 2% agar plate yielded the greatest viability, but this concentration of sucrose was not high enough to counter the internal osmolality and, therefore, not sufficient for injection (Table 1). Taken together, a 2% agar plate covered with 60 mM sucrose dissolved in M4 media provided the best conditions for microinjection of *D. pulex* early embryos, and was employed in subsequent experiments. These conditions allowed embryos to be injected within 30 to 60 min of isolation. It is critical that injections occur within the first hour following ovulation, because there is no cytokinesis during this period and dsRNA can easily diffuse throughout the egg as it remains a single cell [25].

**Table 1:** The relationship between culture conditions and viability (survived juveniles/ total eggs)

	**160 mM (FW)**	**80 mM (FW)**	**60 mM (FW)**	**40 mM (FW)**	**0 mM (FW)**
**Glass petri dish**	0% (0/34)	13.9% (5/36)	45.9% (17/37)	54.1% (20/37)	69.6% (48/69)
**Plastic petri dish**	0% (0/56)	8.5% (5/59)	48.6% (35/72)	59.5% (25/42)	64.8% (35/54)
**2% agar plate**	0% (0/40)	17.9% (7/39)	72.5% (29/40)	65.3% (32/49)	79.2% (38/48)
			**60 mM (M4)**	**40 mM (M4)**	**0 mM (M4)**
**Glass petri dish**			61.9% (26/42)	77.1% (27/35)	88.5% (31/35)
**Plastic petri dish**			62.7% (32/51)	70.0% (28/40)	86.8% (33/38)
**2% agar plate**			88.9% (40/45)	90.9% (40/44)	96.4% (53/55)

Left column represents types of culture plate and top row shows sucrose concentration of culture medium. Sucrose was dissolved in freshwater (FW) or M4 culture medium (M4).

### *Dll* RNAi using microinjection in *D. pulex*

**Figure 1:**
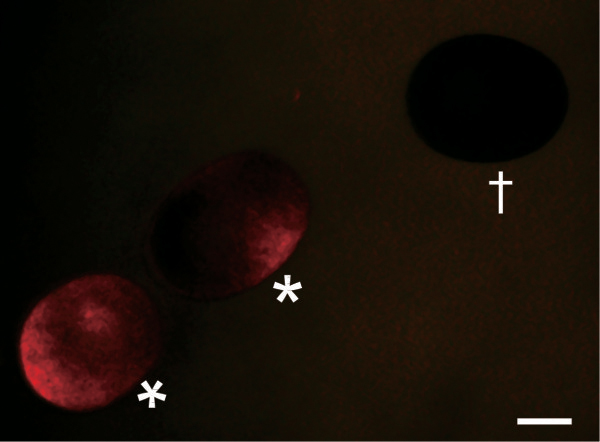
dsRNA-injected (*) and uninjected (†) eggs. Asterisks indicate parthenogenetic eggs just after microinjection of dsRNA with a red fluorescent Chromeo494 dye. Scale bar = 100 μm.

The dsRNA was prepared from a 500 bp region derived from the *Dll* gene (*Dll*-dsRNA; Additional file 2), and dsRNA derived from a 729-bp region of the *Escherichia coli malE* gene (*malE*-dsRNA) was developed as a negative control. There is only one *Dll* gene in the *D. pulex* genome (gene ID: NCBI_GNO_194714, scaffold_121: 199759-203057). The *Dll*-dsRNA was synthesized from a region that included the homeodomain. Red fluorescence was used to judge whether microinjection were successful (Figure 1). Results from RNAi experiments are summarized in Table 2. Only 57% of the embryos injected with *Dll*-dsRNA developed compared to 71.1% viability of those injected with of *malE*-dsRNA, indicating *Dll*-dsRNA produced embryonic lethal phenotypes. Embryonic lethality caused by loss of DLL functions was reported in insects [19,26].

**Table 2:** Summary of RNAi using dsRNA

**dsRNA**	**Injected eggs**	**Juveniles**	**Viability (%)**	**Shortened 2nd antennae**	**Ocellus deletion**
***Dll***	90	51	57	51	34
***malE***	59	42	71.1	0	0

### Sequence-specific gene silencing of *D. pulex Dll*

**Figure 2:**
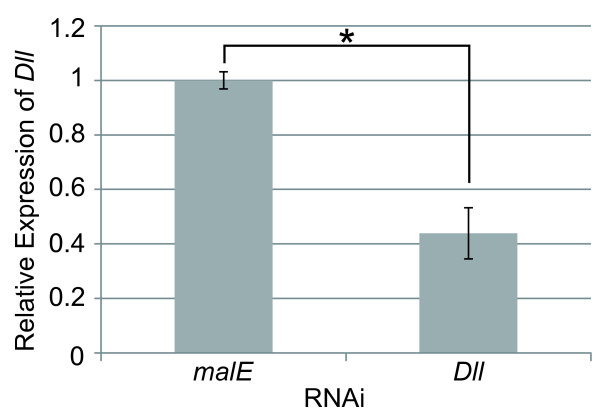
***Dll*expression level in embryos injected with dsRNA of*malE*and*Dll*.** The error bar indicates a mean value ± standard error calculated from triplicate samples. *, value significantly different between *malE* -dsRNA and *Dll*-dsRNA (P < 0.01; *t*-test).

We injected 1 μg/μl dsRNA with a fluorescent dye into each egg and measured the endogenous *Dll* mRNA levels using Q-PCR at 24 h after injection to determine if *Dll*-dsRNA triggers the degradation of endogenous *Dll* mRNAs. The quantity of *Dll* mRNA in *Dll*-dsRNA-injected embryos was decreased to 43.9% (±9.4%) of that in *malE*-dsRNA-injected control embryos (Figure 2). These results were generally consistent with the result of *D. magna* where a 43.6% (±2.5%) reduction in *Dll* was observed [13].

### Phenotype of *Dll* RNAi in *D. pulex*

**Figure 3:**
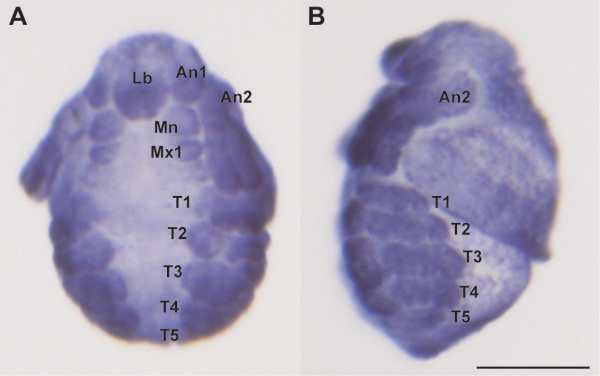
**Spatial expression pattern of*Dll*. A**. Frontal view. **B**. Lateral view. Embryos (around 25-30 h after ovulation) show strong *Dll* signals in first to fifth thoracic limbs, distal portion of second antenna, and labrum, and weak *Dll* signals in first antenna, mandible and first maxillae. An1, first antenna; An2, second antenna; Lb, labrum; Mn, mandible; Mx1, first maxilla; T1-T5, first to fifth thoracic limb. Scale bar = 100 μm.

**Figure 4:**
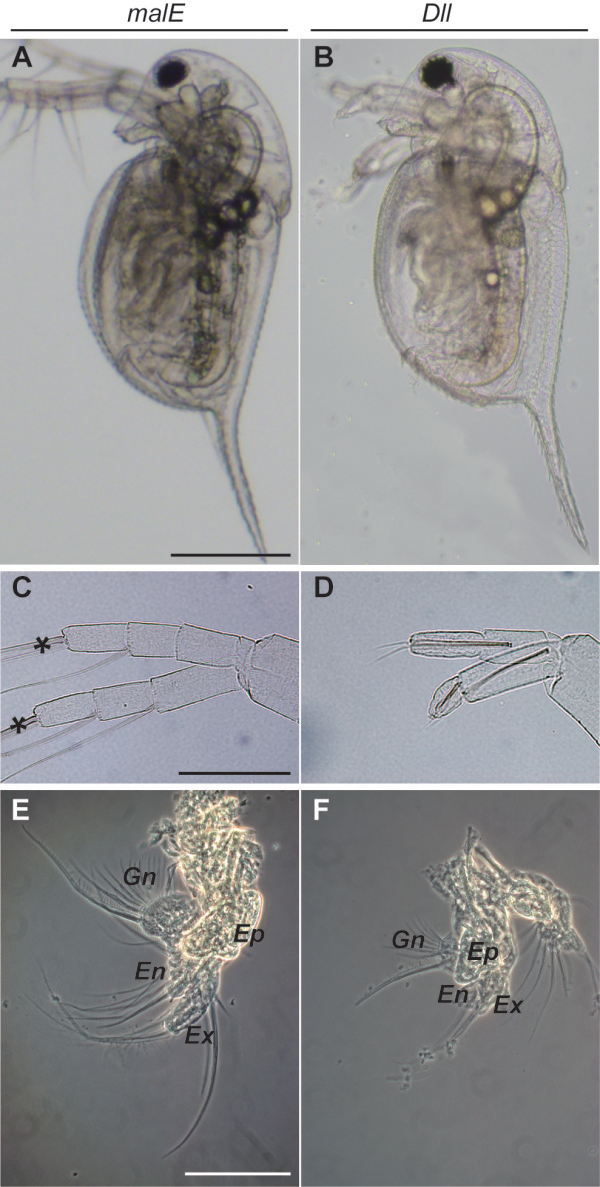
**Major*Dll*-RNAi phenotypes.** The left and right columns show phenotypes of individuals injected with dsRNA of *malE* and *Dll*, respectively. **(A, B)** Lateral view of first instar juvenile. **(C, D)** Lateral view of second antennae. Asterisks indicate the distal portions of the second antennae. **(E, F)** Second thoracic limb (T2). *Ep*, epipodite; *En*, endopodite; *Ex*, exopodite; *Gn*, gnathobase. Scale bars = 200 μm in **A**, **B**; 100 μm in **C**, **D** and **E**, **F**.

We confirmed the spatial expression pattern of *Dll* in embryo using whole-mount *in situ* hybridization. *Dll* was strongly expressed in the distal portion of second antenna, appendages (from the first to fifth thoracic limbs) and labrum, and weakly expressed in first antenna, mandible and first maxilla between 25 to 30 h post ovulation (Figure 3). *Dll*-dsRNAi injection of isolated embryos produced defects in the *Dll*-expressing organs (second antennae, appendages, ocellus, abdominal claw, and abdominal setae) to various degrees. These defects included a truncation of the second antenna segments (Figure 4) that resembled a phenotype observed in *D. magna Dll*-RNAi experiments [13]. The first to fifth thoracic appendages, including each exopodite, were shortened (Figure 4 and Additional file 3). In arthropods truncation of the distal portion of the appendages is one of the well-known phenotypes observed in *Dll* mutation/knockdown [13,18-23]. In addition, a loss of the ocellus and abdominal setae, and a minimized abdominal claw (Additional file 3) were observed in *Dll*-dsRNA injected embryos, which are also concordant with *D. magna*[13]. Although expression of *Dll* in the ocellus has not been reported in any organisms, DLL expression was observed in the prospective ocellus region of *D. magna*[27]. The injection of *non-D. pulex malE*-dsRNA did not induce any morphological abnormalities in *D. pulex* embryos (Figure 3, Additional file 3, and Table 2). These data strongly suggest that the observed phenotypes were specifically generated by *Dll*-dsRNA, mediating the disruption of endogenous *Dll* mRNAs, in *D. pulex*.

## Conclusions

We developed a microinjection system for RNAi in *D. pulex*. The optimum conditions for microinjection were a 2% agar plate with 60 mM sucrose dissolved in M4 media. RNAi can be induced in early embryos by injecting dsRNA into eggs within 1 hour post-ovulation. The microinjection system is applicable to not only RNAi but also creation of transgenic animals, labeling specific target cells, and so on. Thus this technique will contribute *D. pulex* to become a more appropriate species as model organism for environmental, evolutionary, and developmental biology.

## Methods

### *Daphnia* strains and culture conditions

The *D. pulex* were obtained from the Center for Genomics and Bioinformatics (Indiana University, USA) and the National Institute for Environmental Studies (Tsukuba, Ibaraki, Japan). The strains were maintained in dechlorinated freshwater (FW), which was aerated and filtered through activated carbon for 2 weeks, at 18°C under artificial light conditions of 14 h light and 10 h dark to maintain reproduction. A 0.01-ml suspension of 10^9^ cells/ml *Chlorella vulgaris* was added every day to each culture (20-25 individuals/L). For rearing embryos, M4 culture medium (M4) was prepared using MilliQ water [28].

### Culture requirements for isolated eggs

Culture conditions were evaluated to determine the optimum conditions for microinjection. Eggs were dissected from brood chamber just after ovulation using forceps (Dumoxel #5 Biologie, Dumont, Montignez, Switzerland) and placed in a culture plate (glass petri dish, plastic petri dish) containing 2% agar and sucrose (160, 80, 60, 40 mM). Sucrose was dissolved in either FW or M4, as described in the Results and Discussion. These media were passed through a 0.45 μm filter (#431220; Corning, Steuben County, NY, USA) before use. Embryo viability (survived juveniles/total eggs) was evaluated during the first instar juvenile stage.

### Cloning of *Distal-less*

Total RNA was extracted using an ISOGEN kit (NIPPON GENE, Tokyo, Japan), and converted to cDNA using Superscript III and random primers (Life Technologies, Carlsbad, CA, USA) according to the manufacturer’s protocol. The *Dll* fragment was PCR amplified from the cDNA using a set of primers designed from the *Dll* sequence retrieved from wFleaBase http://wfleabase.org/ (Additional file 4). Subsequently, the cDNA fragments were cloned into the pGEM-T vector (Promega, Madison, WI, USA) according to the manufacturer’s instructions. These were sequenced using Sanger techniques that included the Big Dye terminator kit on an ABI 3100 Avant DNA sequencer (Applied Biosystems Japan Ltd, Tokyo, Japan). Two separate clones were developed with same *Dll* region oriented in both directions for dsRNA preparation.

### Whole-mount *in situ* hybridization

*Dll* transcripts were visualized by whole-mount *in situ* hybridization with a *Dll* antisense RNA probe. The 522 bp probes were synthesized using a set of primers (Additional file 4). Embryos at 25-30 h after oviposition were dissected from brood chamber. The samples were transferred into a 1:4 mixture of 4% paraformaldehyde in PBS and heptane for 20 min and rinsed 5 times in PBT (0.1% Tween 20 in PBS). These embryos were digested with proteinase K (4 mg/ml) for 15 min and then rinsed 2 times in PBT. After post-fixation in 4% paraformaldehyde for 20 min, the embryos were rinsed 5 times in PBT and once in a 1:1 mixture of hybridization buffer (HB) and PBST, then transferred to HB at 65°C for at least 2 h. The pre-hybridization buffer was replaced with RNA probe in HB (5 ng/mL) and incubated overnight at 60°C. The digoxigenin (DIG)-labeled RNA probe was prepared from the cloned partial sequences using DIG RNA Labeling Mix (Roche, Mannheim, Germany). Embryos were then rinsed in HB (2:1), 1:1 mixtures of HB and PBST, and washed in PBST 5 times. The DIG hapten was detected with a 1:500 dilution of anti-DIG-alkaline phosphatase antibodies (Roche) in PBST for 1 h at room temperature. The embryo was washed 5 times in PBST and 2 times in the developing solution [0.1 M NaCl, 0.1 M Tris–HCl (pH 9.5), 0.05 M MgCl_2_, 0.1% Tween 20]. The alkaline phosphatase enzyme was detected with NBT/BCIP stock solution (Roche). Embryos were destained in methanol and rehydrated in PBST.

### Preparation of double-stranded RNA

Double-stranded RNA was synthesized using the MEGAscript T7 RNAi Kit (Ambion, Austin, TX, USA). Two templates for the *in vitro* transcription were prepared by PCR using two separate clones (see Cloning of *Distal-less* section) with T7 primer and one side gene-specific primer in each (Additional file 4). The reaction mixes were incubated for 4 h at 37°C, then synthesized sense and antisense RNA were annealed at 75°C for 5 min. This was followed by nuclease digestion to remove DNA and single-stranded RNA, and purification of dsRNA was performed according to manufacturer’s recommended protocol. *malE*-dsRNA was prepared according to methods detailed in Kato et al. [13].

### Injection of double-stranded RNA

The molt of female provides a reference time point of parthenogenetic cycle, which proceeds along a strict time course at 18°C [25]. The female begins to extrude eggs into the brood chamber at 13 min after molting. Eggs were obtained just after ovulation by two week old *D. pulex* and placed in ice-cold M4 medium containing 60 mM sucrose (M4-sucrose). M4-sucrose was passed through a 0.45 μm filter (#431220; Corning) before use. The synthesized dsRNA (2 μg/μl) was mixed with an equal amount of 2 mM Chromeo 494 fluorescent dye (Active Motif Chromeon GmbH, Tegernheim, Germany), which was used as a visible marker for injection. A glass needle was made from a glass capillary tube (GD-1; NARISHIGE, Tokyo, Japan) by a Sutter Instrument (Novato, CA, USA), and the tip was manually cut off using a forceps (Dumoxel #5 Biologie) under a stereomicroscope. A glass petri dish was prepared by placing two cover glasses side by side with M4-sucrose. One egg was placed at the left side cover glass edge to immobilize and injected using an injector (Femtojet, Eppendorf, Hauppauge, NY, USA) and a micro-manipulator (M-152, MMO-220A, Narishige, Tokyo, Japan). The needle was withdrawn from the egg using the right side cover glass edge. Microinjections were carried out within 60 min after ovulation. Then, injected eggs were transferred into a 2% agar in 6-well plate with M4-sucrose and incubated at 18°C. First-instar animals were examined under a stereomicroscope, and their phenotype and viability were recorded.

### Quantitative real-time PCR (Q-PCR)

The relative expression levels of *Dll* mRNAs associated with the two injected conditions (*Dll*-dsRNA and *malE*-dsRNA) were quantified and compared using Q-PCR. Thirteen embryos were collected 24 h following injections with *Dll*-dsRNA or *malE*-dsRNA. Total RNA was extracted and purified using RNAqueous-Micro (Ambion) and converted to cDNA with high capacity cDNA reverse transcription kit (Applied Biosystems) using OligodT primers according to the manufacturer’s recommended protocol. PCR reactions were performed in an ABI Prism 7000 (Applied Biosystems), using SYBR Green I chemistry (Applied Biosystems) in the presence of appropriate primers. The expression stability and suitability of the eight candidate reference genes (GAPDH, aTub, Tbp, Stx16, Xbp1, MMP, CAPON and Actin) were validated employing *geNorm* and *NormFinder* based on Spanier et al. [29]. The actin gene was demonstrated as the most stable reference gene and used for normalization. Primers for both target and control genes were designed to amplify short PCR products of <150 bp (Additional file 4). Data acquisition and analysis were performed by ABI Prism 7000 SDS software ver. 1.1 (Applied Biosystems). The Ct (cycle threshold) was set automatically. Samples included three biological replicates were run in triplicate to capture technical variation, and mean and standard errors calculated by Microsoft Excel 2010. Significant differences between the expression levels of *Dll* and *malE*-dsRNA injected embryos was determined by the *t*-test.

## Competing interests

The authors declare that they have no competing interests.

## Authors’ contributions

All authors designed the experiments; CH, KT and HM performed and analyzed the experiments; CH, JS and TI wrote the paper. KT, HM, YO, NT and SM discussed and commented on results and edited the manuscript. All authors have read and approved the final manuscript.

## Supplementary Material

Additional file 1 **Characteristics of egg in *D. pulex * (A, C) and *D. magna *(B, D).** Samples were collected just after ovulation. (A, B) Light micrographs. (C, D) Hematoxylin and eosin (HE)-stained cross section. od, oil droplet; yg, yolk granule. Scale bar = 100 μm. Click here for file

Additional file 2 **Nucleotide sequences of *Dll-*** **dsRNA.** Click here for file

Additional file 3 **Phenotypes of *Dll*-dsRNA injected juveniles.** The left and right columns show phenotypes of individuals injected with dsRNA of *malE* and *Dll*, respectively. (A, B) First thoracic limb (T1). The exopodite and endopodite were shortened by *Dll*-dsRNA. (C, D) Third and fourth thoracic limbs (T3/4), having the same morphology. The exopodite was shrunk in *Dll*-dsRNA-injected juveniles. (E, F) Fifth thoracic limb (T5). The exopodite was shortened and twisted by *Dll*-dsRNA. (G, H) Lateral view of the rostrum and head. An arrowhead indicates an ocellus. (I, J) Lateral view of abdomens. An arrow and arrowhead show an abdominal claw and abdominal setae. *Ep*, epipodite; *En*, endopodite; *Ex*, exopodite; *Fc*, filter comb. Scale bars = 100 μm. Click here for file

Additional file 4 **Primer sequences for *in situ *** **hybridization, dsRNA and Q-PCR.** Click here for file
