# Supplementary material for: Development of a microinjection system for RNA interference in the water flea Daphnia pulex
Source: BMC Biotechnol. 2013 Nov 5;13:96. doi: 10.1186/1472-6750-13-96 (PMC4228505; doi:10.1186/1472-6750-13-96)

**Additional file 1. Characteristics of egg in *D. pulex* (A, C) and *D. magna* (B, D).**

Samples were collected just after ovulation. (A, B) Light micrographs. (C, D) Hematoxylin and eosin (HE)-stained cross section. od, oil droplet; yg, yolk granule. Scale bar = 100 μm.


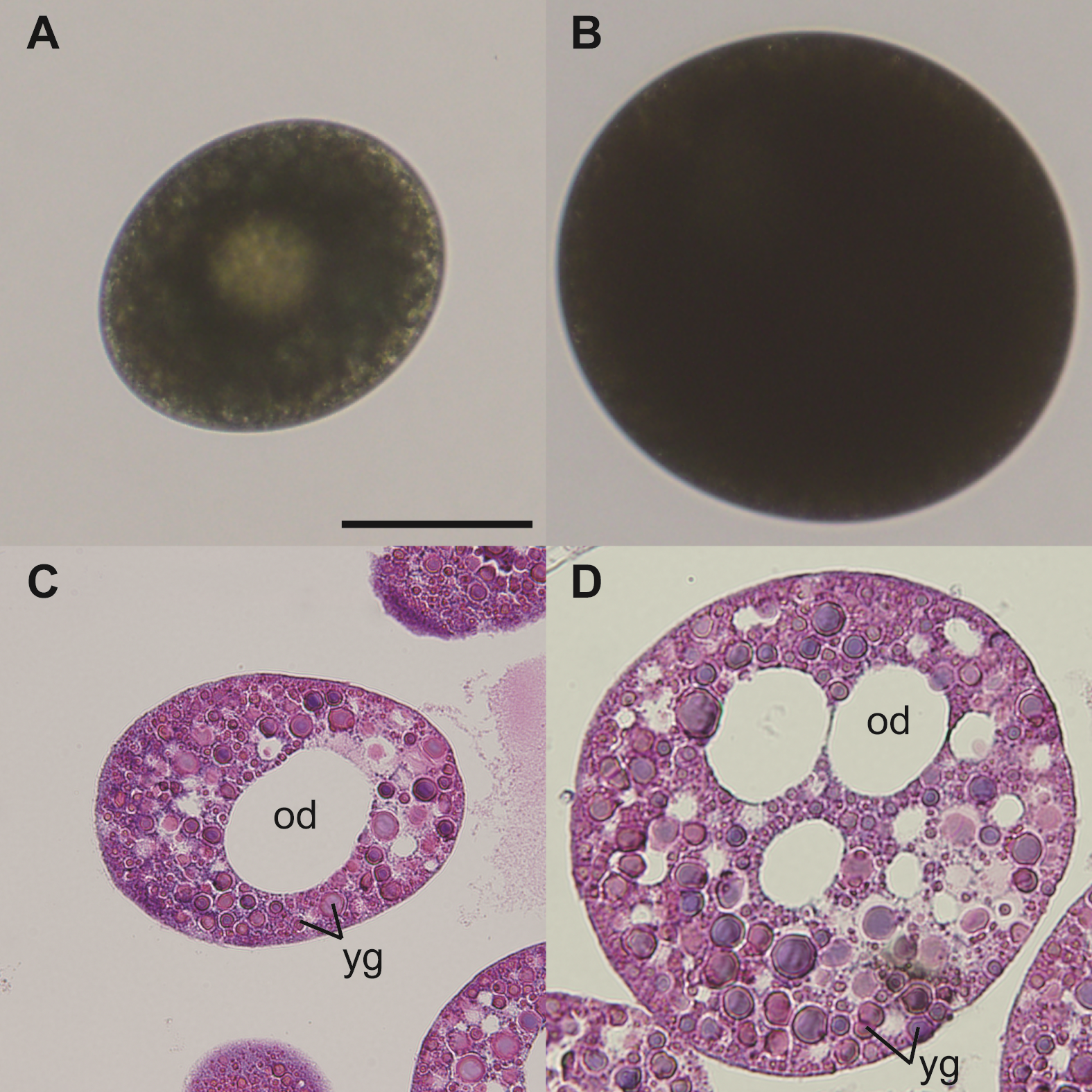

Supplement: Additional file 1 — Characteristics of egg in D. pulex (A, C) and D. magna (B, D). Samples were collected just after ovulation. (A, B) Light micrographs. (C, D) Hematoxylin and eosin (HE)-stained cross section. od, oil droplet; yg, yolk granule. Scale bar = 100 μm. [file 1472-6750-13-96-S1.doc]
