# Supplementary figures and images for: Development of a microinjection system for RNA interference in the water flea Daphnia pulex
Source: BMC Biotechnol. 2013 Nov 5;13:96. doi: 10.1186/1472-6750-13-96 (PMC4228505; doi:10.1186/1472-6750-13-96)

**Additional file 2.** **Nucleotide sequences of *Dll-*dsRNA.**


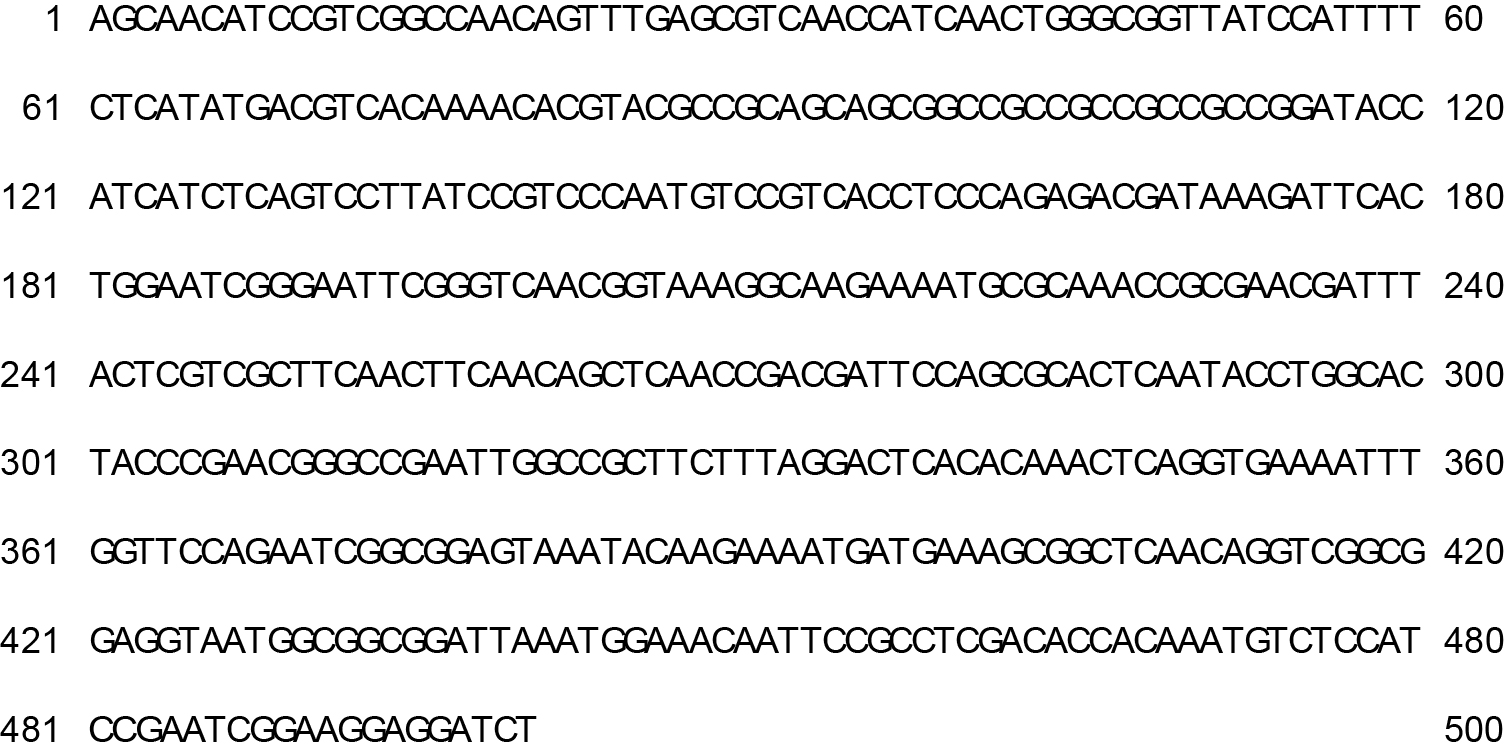

Supplement: Additional file 2 — Nucleotide sequences of Dll- dsRNA. [file 1472-6750-13-96-S2.doc]
