# Supplementary material for: Development of a microinjection system for RNA interference in the water flea Daphnia pulex
Source: BMC Biotechnol. 2013 Nov 5;13:96. doi: 10.1186/1472-6750-13-96 (PMC4228505; doi:10.1186/1472-6750-13-96)

**Additional file 3. Phenotypes of *Dll*-dsRNA injected juveniles.**

The left and right columns show phenotypes of individuals injected with dsRNA of *malE* and *Dll*, respectively. (A, B) First thoracic limb (T1). The exopodite and endopodite were shortened by *Dll*-dsRNA. (C, D) Third and fourth thoracic limbs (T3/4), having the same morphology. The exopodite was shrunk in *Dll*-dsRNA-injected juveniles. (E, F) Fifth thoracic limb (T5). The exopodite was shortened and twisted by *Dll*-dsRNA. (G, H) Lateral view of the rostrum and head. An arrowhead indicates an ocellus. (I, J) Lateral view of abdomens. An arrow and arrowhead show an abdominal claw and abdominal setae. *Ep*, epipodite; *En*, endopodite; *Ex*, exopodite; *Fc*, filter comb. Scale bars = 100 μm.


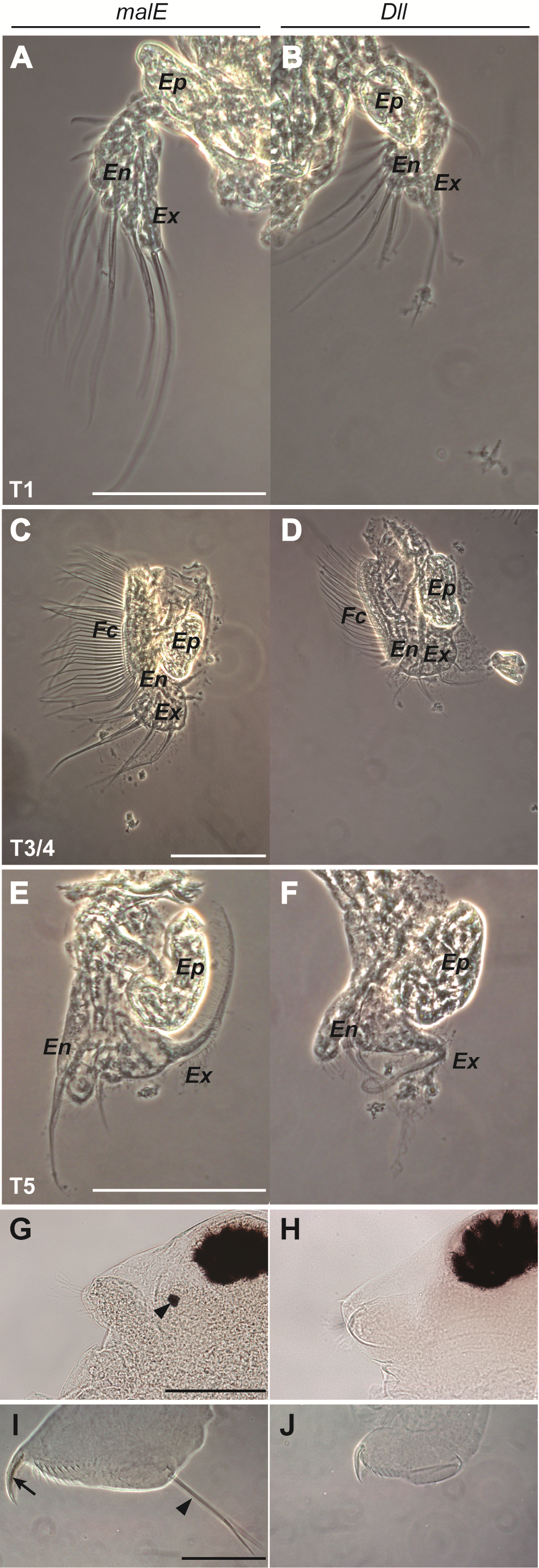

Supplement: Additional file 3 — Phenotypes of Dll-dsRNA injected juveniles. The left and right columns show phenotypes of individuals injected with dsRNA of malE and Dll, respectively. (A, B) First thoracic limb (T1). The exopodite and endopodite were shortened by Dll-dsRNA. (C, D) Third and fourth thoracic limbs (T3/4), having the same morphology. The exopodite was shrunk in Dll-dsRNA-injected juveniles. (E, F) Fifth thoracic limb (T5). The exopodite was shortened and twisted by Dll-dsRNA. (G, H) Lateral view of the rostrum and head. An arrowhead indicates an ocellus. (I, J) Lateral view of abdomens. An arrow and arrowhead show an abdominal claw and abdominal setae. Ep, epipodite; En, endopodite; Ex, exopodite; Fc, filter comb. Scale bars = 100 μm. [file 1472-6750-13-96-S3.doc]
