# Supplementary material for: Development of a microinjection system for RNA interference in the water flea Daphnia pulex
Source: BMC Biotechnol. 2013 Nov 5;13:96. doi: 10.1186/1472-6750-13-96 (PMC4228505; doi:10.1186/1472-6750-13-96)
Supplement: Additional file 4 — Primer sequences for in situ hybridization, dsRNA and Q-PCR. [file 1472-6750-13-96-S4.doc]

**Additional file 4. Primer sequences for *in situ* hybridization, dsRNA and Q-PCR.**

|  | Fwd (5’ to 3’) | Rev (5’ to 3’) |
| --- | --- | --- |
| ***in situ* hybridization** | | |
| Dll | AGCAACATCCGTCGGCC | CGTCGTCTCCGCATTCC |
| ***Dll* cDNA isolation and dsRNA** | | |
| Dll | AGCAACATCCGTCGGCCAACAG | AGATCCTCCTTCCGATTCGG |
| **Q-PCR** | | |
| Actin | GGCTCAGTCCAAGCGTGGTA | TTCCATGTCATCCCAGTTGGT |
| Dll | CATGAGCCGCAATCCTTAC | TCATGTGGGAGAAGGGGTAG |
